# T-Cell Depleted Haploidentical Transplantation in Children With Hematological Malignancies: A Comparison Between CD3+/CD19+ and TCRαβ+/CD19+ Depletion Platforms

**DOI:** 10.3389/fonc.2022.884397

**Published:** 2022-06-20

**Authors:** Marta Gonzalez-Vicent, Blanca Molina, Ivan Lopez, Josune Zubicaray, Julia Ruiz, Jose Luis Vicario, Elena Sebastián, June Iriondo, Ana Castillo, Lorea Abad, Manuel Ramirez, Julian Sevilla, Miguel A. Diaz

**Affiliations:** ^1^ Hematopoietic Stem Cell Transplantation and Cellular Therapy Unit, Hospital Infantil Universitario “Niño Jesus” Madrid, Madrid, Spain; ^2^ Division of Hematology, Blood Bank and Graft Manipulation Unit, Hospital Infantil Universitario “Niño Jesus” Madrid, Madrid, Spain; ^3^ Histocompatibility Lab, Community Transfusion Center of Madrid, Madrid, Spain; ^4^ Oncology/Hematology Lab, Hospital Infantil Universitario “Niño Jesus” Madrid, Madrid, Spain

**Keywords:** haploidentical transplant, hematological malignancies, immune reconstitution, children, T-cell depletion

## Abstract

**Background:**

T-cell depleted (TCD) haploidentical transplantation using CD3+/CD19+ and TCRαβ+/CD19+ depletion techniques has been increasingly used in children with hematological malignancies. We present a retrospective study aimed to compare transplant outcomes in children with leukemia receiving a TCD haploidentical transplant using either CD3+/CD19+ or TCRαβ+/CD19+ platforms.

**Methods:**

A total of 159 children with leukemia (ALL=80) (AML=79) that received a TCD haploidentical transplantation using either CD3+/CD19+ (n=79) or TCRαβ+/CD19+ (n=80) platforms between 2005 and 2020 were included. Median age was 9 years in both groups. There were no differences in patient, donor, and transplant characteristics between groups except for donor KIR B genotype more frequent in the TCRαβ+/CD19+ group (91%) than in the CD3+/CD19+ group (76%) (p=0.009) and a high number of NK+ cells and lower CD19+ cells infused in the TCRαβ+/CD19+ group (35.32x10^6^/kg and 0.06 x10^6^/Kg) than in the CD3+/CD19 group (24.6x10^6^/Kg and 0.25 x10^6^/Kg) (p=0.04 and p=0.0001), respectively. Conditioning was based on TBF. Median follow-up for survivors was 11 years (range; 8-16 y) in CD3+/CD19+ group and 5 years (range; 2-9 y) in the TCRαβ+/CD19+ group.

**Results:**

Engraftment kinetics were similar in both groups (13 days for neutrophils and 10 days for platelets). There was no difference in the incidence of acute GvHD II-IV (29 ± 5% in the CD3+/CD19+ group vs 38 ± 5% in the TCRαβ+/CD19+ group) and chronic GvHD (32 ± 5% vs 23 ± 4%, respectively). NRM was 23 ± 5% in the CD3+/CD19+group vs 21 ± 4% in the TCRαβ+/CD19+group. Relapse incidence was also similar, 32 ± 5% vs 34 ± 6%, respectively. DFS and OS were not different (45 ± 5% vs 45 ± 6% and 53 ± 6% vs 58 ± 6% respectively). As there were no differences on transplant outcomes between groups, we further analyzed all patients together for risk factors associated with transplant outcomes. On multivariate analysis, we identified that early disease status at transplant (HR: 0.16; 95%CI (0.07-0.35) (p=0.0001), presence of cGvHD (HR: 0.38; 95%CI (0.20-0.70) (p= 0.002), and donor KIR-B genotype (HR: 0.50; 95%CI (0.32-0.90) (p=0.04) were associated with better DFS.

**Conclusions:**

Our data suggest that there are no advantages in transplant outcomes between TCD platforms. Risk factors for survival are dependent on disease characteristic, donor KIR genotype, and chronic GvHD rather than the TCD platform used.

## Introduction

Allogeneic hematopoietic stem cell transplantation is a well-stablished therapy for pediatric patients with high-risk hematological malignancies. A human leukocyte antigen (HLA)-identical sibling or an HLA well-matched unrelated donor has been considered the first option for allogeneic transplantation. However, such HLA identical donors are not always available for all patients, or even when having one, they do not have time to be waiting for donor availability (1–5). More than two decades ago, T-cell depleted (TCD) haploidentical transplantation (HaploSCT) was used as an alternative option for hematopoietic stem cell for allogeneic transplantation (6). Biological parents share at least one haplotype with their children and all of them can be considered as potential donors. Moreover, siblings and other relatives might be potential haploidentical donors, which contributes to most pediatric patients having at least one available donor in a timely manner. Although initially the use of haploidentical transplantation was hampered by many clinical complications, the advent of graft manipulation techniques and the use of post-transplant cyclophosphamide (post-Cy) have changed the haploidentical transplant landscape, rapidly increasing its use worldwide over the last few years. As such, HaploSCT is the allogeneic transplant modality and its use has the most growth in recent years not only in adults but also in children (7, 8). Graft manipulation techniques have been used mainly in pediatric patients. They evolved from initial CD34+ selection to the recent CD45RA+ depletion, going by CD3+/CD19+ and TCRαβ+/CD19+ depletion platforms (6, 9–11). Nowadays, several *ex vivo* TCD HaploSCT platforms have gained clinical relevance (12). Considering that there are no prospective studies comparing transplant outcomes of different TCD HaploSCT platforms, data from observational and retrospective studies could be useful in guiding which TCD approach should be chosen. This retrospective, multivariable study was designed to compare transplant outcomes among pediatric patients with hematological malignancies receiving T-cell depletion haploidentical transplant using CD3+/CD19+ or TCRαβ+/CD19+ platforms. We hypothesized that theoretical TCRαβ+/CD19+ advantages in terms of graft composition would result on better transplant outcomes.

## Patients and Methods

### Study Eligibility Criteria, Patients, and Donor Characteristics

All consecutive pediatric patients who received a TCD HaploSCT with CD3+/CD19+ depletion (n=79) or TCRαβ+/CD19+ depletion (n=80) at our institution from January 2005 to January 2020 with hematological malignancies were included in this retrospective study. They were transplanted because of high-risk hematological malignancies and they lacked a suitable HLA-matched (related or unrelated) donor. Indications for allogeneic hematopoietic transplantation for children with acute lymphoblastic leukemia (ALL) included the following: poor cytogenetics, induction failure (defined as no remission at 1 month following induction treatment), persistent MRD with a cutoff point of 10^-3^ and 2^nd^ complete remission (CR) or beyond. For acute myeloid leukemia (AML) patients, transplant criteria included intermediate or poor risk characteristics at diagnosis and also 2^nd^ CR or beyond. Details of transplantation criteria have been published elsewhere (13–15). MRD was performed using multiparametric flow cytometry, following the recommendations of the Euroflow consortium (16) using a FACS Canto II cytometer (BD Bioscience) and combinations of 8 markers per tube. A combination of markers capable of identifying the leukemia-associated immunophenotype (LAIP) was determined for each patient. This combination was used individually at all study times for levels of MRD. For quantitative estimation of MRD levels, a minimum of 500,000 events were acquired in each tube. In hypocellular samples in which the acquisition per tube was <2x10^5^ events, the maximum theoretical sensitivity of the test was indicated (number of LAIP events)/(number of total events acquired) x100), since it did not reach 0.01. At least 20 LAIP events per tube were required for the test to be considered informative.

Patients, donors, parents, and/or their legal guardians gave written informed consent for donation and transplantation in accordance with the Helsinki Declaration. Patients, donors, and transplant characteristics are given in **Table 1**. Donor evaluation included high-resolution HLA typing, eligibility for hematopoietic stem cell donation and mobilization, crossmatch testing, CMV serological status, analysis of Killer-cell Ig-like Receptor (KIR) mismatches, and KIR genotype. Median follow-up for survivors was 11 years (range 8–16 years) for the CD3+/CD19+ group and 5 years (range 2–9 years) for the TCRαβ+/CD19+ group. The last follow-up was on December 2021.

**Table 1:** Patients, donors and transplant characteristics.

Characteristic/Variable	CD3+/CD19+	TCRαβ+/CD19+	P-value
Number of patients	79	80	
Median follow-up of survivors (range), years	11 (8-16)	5 (2-9)	
**Patient-related**			
Age at HCT, median (range), years	9 (1-19)	8.5 (1-19)	n.s.
Gender			
Male/Female	51/28	55/25	n.s.
Lansky score prior to HCT			
60-100	90 (60-100)	90 (60-100)	n.s.
Race			
Caucasian/ non-Caucasians	57/22	57/23	n.s.
CMV recipient/donor pairs n (%):			
Positive/Positive	47 (59)	40 (50)	n.s.
Positive/Negative	7 (9)	13 (16)	
Negative/Positive	18 (23)	16 (20)	
Negative/Negative	7 (9)	11 (14)	
**Disease-related**			
Acute lymphoblastic leukemia:	39 (49)	41 (51)	n.s.
-Disease status at transplant:			
*Early	5 (13)	13 (32)	
*Intermediate	23 (59)	20 (49)	
*Advanced	11 (28)	8 (19)	
			
-MRD status n (%):			
* Positive	12 (31)	6 (15)	
*Negative	27 (69)	35 (85)	
			
-N° of HSCT:			
*1^st^	25 (64)	33 (80)	
*2^nd^	12 (31)	7 (17)	
*3^rd^	2 (5)	1 (3)	
- Phenotype of Acute lymphoblastic leukemia:			
*B	32 (82)	29 (71)	
*T	7 (18)	12 (29)	
Acute myeloblastic leukemia:	40 (51)	39 (49)	n.s.
-Disease status at transplant:			
*Early	12 (30)	18 (46)	
*Intermediate	7 (17)	5 (13)	
*Advanced	21 (53)	16 (41)	
			
-MRD status n (%):			
*Positive	17 (43)	19 (49)	
*Negative	23 (57)	20 (51)	
			
-N° of HSCT:			
*1^st^	27 (67)	27 (69)	
*2^nd^	13 (33)	9 (23)	
*3^rd^	0 (0)	3 (8)	
**Donor-related**			
Age at donation, median (range), years	40 (2-54)	40 (10-54)	n.s.
Gender			
Male/Female	26/53	38/42	n.s.
Donor-recipient relationship n (%)			
Mother	51 (64)	40 (50)	n.s.
Father	21 (27)	31 (39)	
Sibling	7 (9)	9 (11)	
KIR disparity: Yes/No	44/35	47/33	n.s.
KIR genotype: B /A	60/19	73/7	**0.009**
**Transplant-related**			
Conditioning regimen n (%)			
TBF	65 (82)	68 (85)	n.s.
Flu-Mel	14 (18)	12 (15)	
Previous transplant			
0	49 (62)	60 (75)	n.s.
1	28 (36)	16 (20)	
2	2 (2)	4 (5)	
**Graft composition**			
CD34+ cells (x10^6^/kg)	7.28 (1.19– 41.6)	8.67 (2.06-55.8)	0.08
CD3+ cells (x10^5^/kg)	0.1 (0.01-11.8)	77.11 (6.97-640.92)	
αβ T cells (x10^6^/Kg)		0.025 (0-0.078)	
γδ T cells (x10^6^/Kg)		6.84 (2.5-64.1)	
NK cells (x10^6^/kg)	24.6 (2.57-141.3)	35.32 (2.77-177)	0.004
B cells (x10^6^/kg)	0.25 (0-33.36)	0.06 (0-1.34)	0.001

CMV, cytomegalovirus; KIR, Killer-cell Ig-like Receptor; MRD, Minimal Residual Disease; CR, Complete Remission; TBF, Thiotepa, Busulfan, Fludarabine; Flu-Mel, Fludarabine, Melphalan; n.s, not significant. The bold values are the statistically significant values.

### Donor Hematopoietic Stem Cell Mobilization and Collection Procedures

Donor mobilization was performed as previously described (13–15). Briefly, donor peripheral blood stem cell progenitors were mobilized by administering subcutaneous granulocyte colony-stimulating factor (10 μg/kg per day for 4 days) and were harvested by 1 or 2 large-volume leukapheresis procedures according to established protocols of the center using a continuous flow blood cell separator (Spectra OptiaMNC v.3.0. Terumo BCT, Lakewood, CO; USA or COBE Spectra TM, v.6.1, by Caridian BCT Europe, Garching, Germany) on the fifth day of mobilization and the day before infusion.

### Graft Manipulation and Infusion Procedures

T-cell depletion was performed using CliniMACS Plus device or the fully automated Prodigy device after manipulations in a laminar-flow cabinet located in a clean room certified for sterile manipulations. All immuno-magnetic procedures were performed according to the manufacturer’s standard protocol. Clinical grade reagents, disposable kits, and instrumentation were from Miltenyi Biotec (Bergisch Gladbach, Germany). Before and after depletion procedures, the apheresis, and final product cell composition were analyzed by flow cytometry (cell count and cell subpopulation CD34+, CD3+ αβ+, CD3+ γδ+, CD19+, and CD3-CD56+ cells, and their viability). Finally, the cell product was freshly infused *via* a central venous catheter on day 0.

### KIR Ligand and KIR Genotyping Analysis

The KIR ligand HLA-C allotypes (C1 and C2) and the HLA-B allotypes (Bw4) were determined using high-resolution PCR-sequence-based typing. Fifteen human KIR genes and two pseudogenes were analyzed by PCR with a KIR genotyping kit (Miltenyi Biotec, Bergisch Gladbach, Germany). The KIR A haplotype was defined by the absence of 2DS1, 2DS2, 2DS3, and 3DS1 and the presence of 2DS4 as the only KIR-activating receptor. The KIR B haplotype was determined by the presence of any activating genes except 2DS4 (17). We determined KIR B-content scores for all donors according to the system proposed by Cooley et al. (18) (www.ebi.ac.uk/ipd/kir/donor_b_content.html). Criteria for donor selection have been previously published (19). Donor characteristics are shown in **Table 1**.

### Conditioning Regimen and Pharmacological GvHD Prophylaxis

The conditioning regimens consisted of intravenous fludarabine at 30 mg/m^2^/day for 5 days (day -6 to day -2), intravenous busulfan administered once daily according to patient body weight for 3 days (day -5 to day -3), intravenous thiotepa at 5 mg/kg/day for 2 days (day -3 to day -2), and intravenous methylprednisolone at 4 mg/kg/day (day -6 to day -2). Busulfan weight-based dosing was as follows: <9 kg: 1 mg/kg/dose; 9–16 kg: 1.2 mg/kg/dose; 16–23 kg:1.1 mg/kg/dose; 23–34 kg: 0.95 mg/kg/dose, and >34 kg: 0.8 mg/kg/dose (20). For those patients who needed a second haploidentical transplantation due to graft failure (21), the conditioning regimen consisted of fludarabine 40 mg/m^2^/day from days −5 to − 3, thymoglobulin 2 mg/kg/day from days −5 to − 3, and melphalan 120 mg/m^2^ on days -1 to -2. Cyclosporine was used as pharmacological GvHD prophylaxis from day −1 until engraftment and it was tailored after transplant while acute GvHD was not present.

### Study Design, Endpoints, Definitions, and Statistical Analysis

This study was designed to retrospectively compare transplant outcomes between two TCD haploidentical transplant cohorts. Primary endpoints were disease-free survival (DFS), relapse incidence (RI), and non-relapse mortality (NRM). Secondary endpoints were engraftment kinetics and early and late immune reconstitution. Graft-versus-Host disease (GvHD) and infections incidence were also analyzed. GvHD was diagnosed and graded according to previously described definitions (22–24). Phenotyping of NK cells, T lymphocytes, T lymphocyte subsets, and B lymphocytes was performed on fresh samples of whole blood by multi-parametric flow cytometry as previously described (25). Immune reconstitution was analyzed at day +15, +30, +60, +90, +180, +270, +1 year, and 2 years following transplantation. DFS was defined as time from transplantation to either relapse or death of any cause. Relapse was defined as morphological or clinical evidence of recurrence in peripheral blood, bone marrow, or extramedullary sites. NRM was defined as any cause of death other than relapse. Categorical variables were summarized as frequencies and percentages. Continuous variables were presented as medians and ranges. Comparison of two means has been made with the non-parametric Mann-Whitney U test. Cumulative incidence was used to estimate the relapse and NRM probabilities (26). Death in remission was treated as a competing event to calculate the cumulative relapse incidence. Relapse was considered to be the competing event for calculating cumulative incidence of NRM. Gray’s test was used to assess differences between relapse incidence and NRM. Probabilities of OS, LFS, and EFS were calculated according to the Kaplan and Meier method. The significance of differences between survival probabilities was estimated by the log-rank test (Mantel-Cox). The multivariate analysis of survival was evaluated using a proportional hazard regression model (27). Hazard ratios (HRs) were calculated with a 95% confidence interval (95% CI). The following variables were included in the analysis as covariates: patient and donor age, diagnosis, disease and MRD status at the time of transplantation, cell graft composition, KIR ligand mismatch status, donor KIR haplotype, KIR B score, graft versus host diseases and kinetics of immune reconstitution. Patients were censored at the time of last contact, loss of follow-up, or death. A p value <0.05 was considered statistically significant. Statistical analyses of data were performed using the statistical package SPSS for Macintosh (software version 20.0; IBM Corporation, Armonk, NY, USA) and the R software package for Macintosh (version R 3.3.3).

## Results

### Hematopoietic Engraftment, Supportive Care, and Clinical Toxicity Profile

There were no statistical differences in neutrophil and platelets engraftment between groups. The median times to neutrophil recovery were 13 days (range: 8–28) and 13 days (range: 10–26) for the CD3+/CD19+ and the TCRαβ+/CD19+ groups, respectively. Median time to platelet recovery (20 x10^3^/µL) and (100 x10^3^/µL) were 10 days (range: 6-40) and 15 days (range; 6-270) for CD3+/CD19+ group and 9 days (range: 6-27) and 13 days (range: 9-153) for the TCRαβ+/CD19+ group (p=0.07).

Primary graft failure occurred in 10 out of 79 patients (12%) in the CD3+/CD19+ group and 8 out of 80 (10%) in the TCRαβ+/CD19+ group. This difference was not statistically significant. Median age of patients with graft failure was 5 years (1-9), 14 were male. There were 7 patients (39%) diagnosed with ALL and 11 (61%) with AML. Half of them were in the early phase of the disease. Nine patients (9/18) received a second transplant and five are alive. Six patients had an autologous reconstitution and are in complete remission and three patients relapsed.

The median length of hospitalization was 15 days (range: 10-127) in the CD3+/CD19+ group and 15 days (range 10–203) in the TCRαβ+/CD19+ group. Patients in the CD3+/CD19+ group received red blood transfusions for a median time of 2 days (range: 0–21) and similarly in the TCRαβ+/CD19+ group: 2 days (range: 0–24)). The platelets transfusion was also similar between groups: 3 days (range: 0–40) for the CD3+/CD19+ group and 2 days (range: 0–54)) for the TCRαβ+/CD19+ group. The median of antibiotic treatment days was 13 (range: 0–122) in the CD3+/CD19+ group and 17 days (range: 3–80) in TCRαβ+/CD19+ group (p=0.003). In the CD3+/CD19+ group, the median time needing parenteral nutrition support was 7 days (range; 0-20) compared with 0 days (range: 0-26) in the TCRαβ+/CD19+ group (p=0.09)).

Nausea and vomiting, diarrhea, and hemorrhagic cystitis were the most common toxicities in both groups. Twelve out of 80 patients developed VOD in the TCRαβ+/CD19+ group, whereas only 2 out of 79 did in a CD3+/CD19+ group (p=0.006). Data regarding toxicity profile are provided in **Supplementary Table S1**.

### Infections

In all, 149 infectious episodes were diagnosed using microbiological and/or clinical criteria in the CD3+/CD19+ group and 170 in the TCRαβ+/CD19+ group. All patients suffered some kind of infection during transplant admission. Taking together all infection episodes, there were no significant differences in the incidence of infection complications between groups. Viral infections were the most frequent ones in both groups (52% in the CD3+/CD19+ group vs 63% in the TCRαβ+/CD19+ group). However, there was a trend to higher incidence of viral infections in the TCRαβ+/CD19+ group with a higher incidence of HHV-6 in the TCRαβ+/CD19+ group, whereas VZV infection was more frequent in CD3+/CD19+ group.

HHV-6 reactivation occurred in 23 patients and eleven developed encephalitis diagnosed by neurological symptoms, positive PCR for HHV-6 in cerebrospinal fluid, and the absence of other identified etiologies of CNS dysfunction. Main neurological symptoms were alteration of consciousness, amnesia, seizures, hypothermia, hyponatremia, and pruritus. Treatment was mainly based in foscarnet. One patient died by HHV6 disseminated disease.

Bacterial infections were the second group of infections comprising 39% of all in the CD3+/CD19+ group and 29% in the TCRαβ+/CD19+ group. However, there was a higher incidence of *Clostridium species* in the TCRαβ+/CD19+ group (30% of bacterial episodes) compared with 12% in the CD3+/CD19+ group. Detailed data regarding bacterial, viral, and fungal infections are provided in **Supplementary Table S2**.

### Immune Reconstitution

Immune reconstitution in the early phase after transplant was characterized in both groups by a rapid increase in NK cells on day +15 (median; 204/µL, range; 2–1840 in CD3+/CD19+ group and 172/µL, range: 1-977 in the TCRαβ+/CD19+ group) and specially on day + 30 (median: 285/µL, range: 1–1895 in the CD3+/CD19+ group and 297/µL, range: 0-3192 in TCRαβ+/CD19+ group). These differences were not statistically significant.

T cells and their subsets progressively increase since day +60 in both groups become the predominant lymphocyte population on day +90 and afterward. There was a trend for a better B-cell reconstitution in the TCRαβ+/CD19+ group from day +270. Detailed data on absolute lymphocyte count (ALC), NK cells, T lymphocytes, T lymphocyte subsets, and B lymphocytes following transplant are provided in **Supplementary Table S3**.

### Acute and Chronic Graft Versus Host Disease

Thirty-one patients developed aGvHD in the CD3+/CD19+ group (grade I: 13, grade II: 8, grade III: 7 and grade IV: 3) and 40 (grade I: 13, grade II: 12, grade III: 8 and grade IV: 7) in TCRαβ+/CD19+ group. The median time to aGvHD was 30 days (range, 10–90 days) in the CD3+/CD19+ group and 29 days (range, 10–95 days) in the TCRαβ+/CD19+ group. The cumulative incidence of aGvHD was 29±5% in the CD3+/CD19+ group and 38±5% in the TCRαβ+/CD19+ group. These differences were not statistically significant. Acute GvHD treatment in the first line was based on steroids and in the second line ruxolitinib and immunomodulatory therapy with photopheresis and mesenchymal cells. GvHD was the main cause of death in 7 patients.

Twenty-three patients developed cGvHD (mild: 10, moderate: 11, and severe: 2) in the CD3+/CD19+ group and 17 (mild: 6, moderate: 7, and severe: 4) in the TCRαβ+/CD19+ group. The median time to cGvHD was 120 days (range: 41–306 days), in the CD3+/CD19+ group and 119 days (range: 45–454 days) in the TCRαβ+/CD19+ group. The cumulative incidence of cGvHD was 32±5% in the CD3+/CD19+ group and 23±4% in TCRαβ+/CD19+ group. These differences were not statistically significant.

### Non-Relapse Mortality

The cumulative incidence of NRM was 23±5% in the CD3+/CD19+ group. Seventeen patients died with a median time to death of 103 days (range: 30-1391 days). In the TCRαβ+/CD19+ group, the cumulative incidence of NRM was 21±4%. Fifteen patients died with a median time to death of 86 days (range: 27-543 days). The leading cause of death was relapse of disease in both (21% and 19%, respectively) followed by infection (9% and 14%, respectively). These differences were not statistically significant. The primary causes of death are given in **Table 2**.

**Table 2:** Causes of death.

	CD3+/CD19+ (n=32)	TCRαβ+/CD19+ (n=28)	P value
Relapse	15	13	n.s.
Infection:	7	4	n.s.
• Adenovirus	0	1	
• CMV	2	0	
• EBV	0	1	
• HHV6	0	1	
• RSV	1	0	
• Rubeola	1	0	
• VZV	1	0	
• Pseudomonas spp	0	1	
• Aspergillus	2	0	
GvHD	3	4	n.s.
TA-TMA	1	4	n.s.
Graft failure	1	1	n.s.
VOD/SOS	1	1	n.s.
Other	4	1	n.s.

CMV, cytomegalovirus; EBV, Epstein Barr virus; HHV6, human herpes virus 6; RSV, respiratory syncytial virus; GvHD, Graft-versus-Host Disease; TA-TMA, Transplant Associated Thrombotic Microangiopathy; VOD/SOS, Veno-Occlusive Disease/Sinusoidal Obstruction Syndrome; n.s, not significant.

### Relapse

Twenty-three patients relapsed in the CD3+/CD19+ group and twenty-four in the TCRαβ+/CD19+ group. Median time to relapse was 164 days (range: 23-1286 days) in CD3+/CD19+ group and 206 days (range: 37-1876) in the TCRαβ+/CD19+ group. The cumulative incidence of relapse was 32±5% in CD3+/CD19+ group and 34±6% in the TCRαβ+/CD19+ group. This difference was not statistically significant. Nine patients underwent a second hematopoietic transplant after relapse treatment and four are alive and in complete remission.

### Disease-Free Survival and Overall Survival

Probability of DFS was 45±5% in the CD3+/CD19+ group and 45±6% in the TCRαβ+/CD19+ group. Probability of OS was 53±6% and 58±6% for CD3+/CD19+ patients and TCRαβ+/CD19+ patients, respectively. These differences were not statistically significant.

We analyzed the patients transplanted after a previous allograft and whose OS was also similar: 60± % after the 1^st^ transplant and 47 ±7% beyond (p=ns).

### Secondary Risk Factors Analysis

Considering that there were no differences in transplant outcomes between both groups, we analyzed risk factors associated to transplant outcomes for the whole group. Univariate analysis (UVA) showed that NRM was associated with the following variables: disease status at transplant (9±4% in 1^st^ CR, 28±6% in 2^nd^ CR, and 27±6% in >2^nd^ CR; p=0.005); amount of NK cells on day +30 (11±4% in patients with ≥ 300/µL NK cells and 30±5% in patients with < 300/µL NK cells; p=0.005), and donor kinship (6±4% with sibling donors and 24±3% with parents as donors; p=0.09). However, in multivariate analysis (MVA), disease status at transplant and the amount of NK cells on day+30 were the variables associated with NRM (**Table 3**). In UVA, relapse incidence was associated with the disease status at transplant (13±5% in 1^st^ CR, 38±6% in 2^nd^ CR, and 40±6% in >2^nd^ CR; p=0.004); MRD status at transplant (27±4% in MRD negative and 43±7% in MRD positive patients; p=0.03): donor KIR genotype (27±4% in KIR B genotype and 52±10% in KIR A p=0.01), and cGvHD (20±6% in patients with cGvHD and 37±4% in patients without cGvHD; p=0.03). However, relapse was associated with disease status and cGvHD in MVA (**Table 3**).

**Table 3:** MVA of transplant outcomes for whole group (n= 159).

Variables	HR	95% CI lower limit	95% CI upper limit	P value
**NRM**				
**Disease status at transplant**				
1^st^ and 2^nd^ CR	1			
>2^nd^ CR	4.2	1.19	14.98	**0.02**
**NK cells day+30**				
≥ 300/µL	1			
< 300/µL	4.16	1.65	10.20	**0.002**
**Relapse**				
**Disease status at transplant**				
1^st^ CR	1			
2^nd^ CR	3.3	1.12	9.73	**0.03**
>2^nd^ CR	6.2	2.06	18.39	**0.001**
**cGvHD**				
Yes	1			
No	3.12	1.42	6.83	**0.005**
**DFS**				
**Disease status at transplant**				
1^st^ CR	1			
2^nd^ CR	0.5	0.32	0.91	**0.02**
>2^nd^ CR	0.2	0.09	0.48	**0.0001**
**cGvHD**				
Yes	1			
No	0.4	0.23	0.75	**0.004**
**KIR Genotype**				
B	1			
A	0.5	0.32	0.90	**0.04**

MVA, Multivariate Analysis; HR, hazard ratio; CI, confidence intervals; DFS, Disease-free survival; NRM, Non-relapse mortality; cGvHD, chronic Graft-versus-Host Disease; CR, complete remission.The bold values are the statistically significant values.

**Figure 1:**
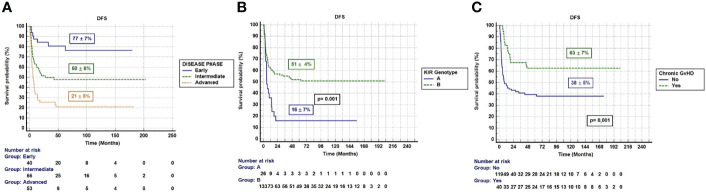
**(A)** DFS according disease phase (Early: 1st CR, Intermediate: 2nd CR, Advanced: >2nd CR or active disease). **(B)** DFS according donor KIR Genotype. **(C)** DFS according chronic GvHD.

UVA showed that DFS was influenced by the following pre-transplant variables: disease status at transplant (77±7% in 1^st^ CR, 50±6% in 2^nd^ CR, and 21±5% in >2^nd^ CR; p=0.0001); MRD status at transplant (53±5% in MRD negative and 30±5% in MRD positive patients; p=0.001), donor KIR genotype (57±4% in KIR B genotype and 16±7% in KIR A p=0.001), and donor kinship (74±11% with sibling donors and 41±4% with parents as donors; p=0.04). DFS was also influenced by the following post-transplant variables: NK cells on day+30 (52±6% in patients with ≥ 300/µL NK cells and 42±5% in patients with < 300/µL NK cells; p=0.05) and chronic GvHD (63±7% in patients with cGvHD and 38±5% in patients without cGvHD; p=0.001). In MVA, variables that impact on DFS were disease phase at time of transplantation, the presence of chronic GvHD, and donor KIR genotype (**Table 3**). Patients transplanted in the advanced phase of disease, patients with no chronic GvHD, and those transplanted from a donor KIR A genotype had a reduced probability of DFS [HR; 0.16, 95% CI: 0.07-0.35; p=0.0001), (HR; 0.38, 95% CI: 0.20-0.70 p=0.002) and (HR; 0.50, 95% CI: 0.32-0.90 p=0.04)] (**Table 3**) and (**Figure 1**).

## Discussion

Nowadays, allogeneic hematopoietic stem cell transplantation from haploidentical donors is a suitable option for those pediatric patients lacking a HLA-identical donor. The advent of T-cell depletion techniques has made possible a wider use of haploidentical transplant in pediatric patients. Over the last two decades, we have been witnesses of a rapid evolution in T-cell depletion techniques. These techniques have evolved from the initial almost total T and B cells depletion by means of CD34+ isolation and CD3+/CD19+ depletion to more recently graft manipulation techniques such as TCRαβ+/CD19+ or CD45RA+ depletion that resulted to partial TCD holding back NK and some T-cell subset (γδ and memory T cells, respectively) in the graft (12, 28–30). TCRαβ+/CD19+ depletion method that retains γδ T cells in the inoculum has theoretical advantages over CD3+/CD19+ depletion because γδ T-cells in the graft could improve anti-infectious and anti-leukemic effect. However, there are neither prospective nor retrospective reported studies comparing both options and it is not clear which one would be the better option. Therefore, the primary objective of this analysis was to compare DFS and other transplantation outcomes after haploidentical donor transplantation using CD3+/CD19+ or TCRαβ+/CD19+ depletion procedures.

To our knowledge, this is the first study comparing both TCD procedures in pediatric haploidentical transplant setting. Our results clearly suggest that there are no relevant differences in terms of transplant outcomes between the two groups of patients. Patients’ characteristics are similar between groups for the most potential variables affecting transplant outcomes. It is noteworthy that in both groups there is a high proportion of patients transplanted in the advance phase of disease (more than 20%) and with MRD positive (more than 30%) at the time of transplant. Disease status at transplant is associated not only with relapse incidence but also with NRM and obviously importantly impacts on DFS. Considering that disease status and MRD status at transplant are the most important well-known variables influencing the transplant outcome, this might explain the absence of differences between groups.

Engraftment kinetics are also similar in both groups, although a trend to faster platelet engraftment is observed in TCRαβ+/CD19+ likely mirroring the higher number of CD34+ cells infused in that group of patients. Graft failure incidence is also similar in both groups with a very low impact on the cause of death in both groups. Beyond HLA disparity, there are other potential risk factors for graft failure in the TCD haploidentical transplant. Recently, patient age and the pre-transplant number of CD3+CD8+ cells have been reported as risk factors in the TCD haploidentical transplant setting (21). The use of serotherapy, as part of conditioning regimen, might increase the probability of primary engraftment but increase the risk of delayed immune reconstitution (31).

The toxicity profile is very similar in both owing to the fact that there were no differences in the conditioning regimen used. However, the higher incidence of SOS/VOD in TCRαβ+/CD19+ group is remarkable. There is no clear explanation for this finding as there are no differences in race/ethnicity or intensity of conditioning between groups, but it may be because the new broader diagnostic criteria of VOD in children proposed by EBMT caused a real increase in VOD incidence. It is important to the use of Busulfan TDM to decrease the incidence of VOD after transplant. Despite this, SOS/VOD has no important impact as the cause of death in both groups maybe due to defibrotide treatment (32).

Early phase of immune reconstitution is dominated by a rapid rise of NK cell population in both groups with no differences (13, 15, 25). T-cell reconstitution kinetics are also similar but a better CD4+ cells and B cells reconstitution is observed in the TCRαβ+/CD19+ group by 9 months after transplant. However, it does not seem to impact on infection complication likely due to the fact that most of viral and bacterial complication occurred very early following transplantation when a deep lymphopenia happens in both groups of patients. Nevertheless, the high incidence of HHV-6 infections in both groups but especially in the TCRαβ+/CD19+ group is remarkable (33, 34). HHV-6, a T-lymphotropic herpesvirus, can productively infect γδ T lymphocytes, inducing important phenotypic and cytopathic changes. Thus, by directly attacking γδ T cells, HHV-6 may seek to escape the immune control of the host and thereby establish persistent infection (35). This may be the reason of increased incidence for HHV6 with αβ depletion.

NK cells reconstitution in the early phase after transplant has been considered to have an impact on transplant outcomes (13, 15, 36, 37). A rapid NK cell reconstitution may prevent infections complications. However, a high incidence of CMV reactivation and other viral complications in haploidentical transplant setting especially when using post-Cy as GvHD prophylaxis has been published (38, 39). Infections are the main cause of death apart from relapse in the whole group and still continue being an unsolved clinical problem. As expected, NRM is clearly associated with disease status at transplant. A rapid NK cell recovery is associated with a reduction on NRM because of a trend of lower incidence of severe GvHD.

Relapse after transplant still represents the main cause of transplant failure. Several mechanisms of immune evasion have been described such as genomic loss of HLA, a relapse modality first described in haploidentical transplant, the transcriptional downregulation of HLA class II molecules, and the enforcement of inhibitory checkpoints between T cells and leukemia (40). As expected, disease status at transplant and cGvHD are both associated with relapse incidence in MVA as previously described (13, 15).

NK cells can also exert a notable GvL effect with no GvHD. However, it is not clear whether this GvL effect is mediated by NK cell alloreactivity based on donor/recipient KIR mismatching or by the presence of KIR activating receptors in donors with KIR-B genotype (15, 41). Likely, the use of T-cell depletion or post-Cy as GvHD prophylaxis plays a key role on NK cell reconstitution and the NK cell associated GvL (42, 43).

Finally, the MVA of transplant outcomes shows that patients transplanted in advanced phase of disease and those transplanted from a donor with KIR A genotype, had worse DFS due to a very high risk of relapse and mortality. Conversely, those patients that developed chronic GvHD did better in terms of DFS due to lower relapse incidence, no matter the transplant group. We acknowledge several limitations to the present study. The study limitations are mainly those derived from its retrospective nature. It covers a broad time range during which there have been several major changes in this field. In addition, being TCRαβ+/CD19+ depletion a more recent procedure, the follow-up in these patients was shorter than those transplanted with CD3+/CD19+ depletion procedure. Notwithstanding limitations inherent in all retrospective studies, we believe that the present study provides a valuable information to the hematopoietic transplant community regarding risk factors for transplant outcomes among pediatric patients receiving TCD haploidentical transplant.

Maybe in the next future, combination of myeloablative conditioning with T regulatory and T conventional cells as immunotherapy might reduce relapse incidence with no increase of risk of severe GvHD (44, 45).

In conclusion, the study suggests that there are no advantages in transplant outcomes between both TCD platforms. Risk factors for survival are clearly dependent on disease characteristic, donor KIR genotype, and chronic GvHD rather than the TCD platform used.

## Data Availability Statement

The original contributions presented in the study are included in the article/**Supplementary Material**. Further inquiries can be directed to the corresponding author.

## Ethics Statement

The studies involving human participants were reviewed and approved by Ethics committee of Hospital Niño Jesus. Written informed consent to participate in this study was provided by the participants’ legal guardian/next of kin.

## Author Contributions

MG-V is responsible for caring patients and statistical analysis and wrote the manuscript. BM cared for the patients. IL cared for the patients and collected data. JZ, ES, JI, and JS performed the graft manipulation. JR collected the data. JV performed the HLA study. AC, LA, and MR performed the cytometry analysis. MD wrote the manuscript. All authors contributed to the article and approved the submitted version.

## Conflict of Interest

The authors declare that the research was conducted in the absence of any commercial or financial relationships that could be construed as a potential conflict of interest.

## Publisher’s Note

All claims expressed in this article are solely those of the authors and do not necessarily represent those of their affiliated organizations, or those of the publisher, the editors and the reviewers. Any product that may be evaluated in this article, or claim that may be made by its manufacturer, is not guaranteed or endorsed by the publisher.
