# Correction: T-cell depleted haploidentical transplantation in children with hematological malignancies: a comparison between CD3+/CD19+ and TCRαβ+/CD19+ depletion platforms

**DOI:** 10.3389/fonc.2025.1680867

**Published:** 2025-09-08

**Authors:** Marta Gonzalez-Vicent, Blanca Molina, Ivan Lopez, Josune Zubicaray, Julia Ruiz, Jose Luis Vicario, Elena Sebastián, June Iriondo, Ana Castillo, Lorea Abad, Manuel Ramirez, Julian Sevilla, Miguel A. Diaz

**Affiliations:** ^1^ Hematopoietic Stem Cell Transplantation and Cellular Therapy Unit, Hospital Infantil Universitario “Niño Jesus” Madrid, Madrid, Spain; ^2^ Division of Hematology, Blood Bank and Graft Manipulation Unit, Hospital Infantil Universitario “Niño Jesus” Madrid, Madrid, Spain; ^3^ Histocompatibility Lab, Community Transfusion Center of Madrid, Madrid, Spain; ^4^ Oncology/Hematology Lab, Hospital Infantil Universitario “Niño Jesus” Madrid, Madrid, Spain

**Keywords:** haploidentical transplant, hematological malignancies, immune reconstitution, children, T-cell depletion

In the published article, there was a mistake in the “Author contributions” of the article. The author Ivan Lopez contribution was erroneously given as “second author”. Author Ivan Lopez shares first authorship and contributed equally with Marta Gonzalez-Vicent and Blanca Molina. The correct author contributions statement is “These authors have contributed equally to this work and share first authorship”.

The original version of this article has been updated.

