# Supplementary material for: T-Cell Depleted Haploidentical Transplantation in Children With Hematological Malignancies: A Comparison Between CD3+/CD19+ and TCRαβ+/CD19+ Depletion Platforms
Source: Front Oncol. 2022 Jun 20;12:884397. doi: 10.3389/fonc.2022.884397 (PMC9251308; doi:10.3389/fonc.2022.884397)
Supplement: Supplementary file 1 [file Table1.docx]

**Supplementary Table S1. Toxicity profile.**

| **Variable** | **CD3+/CD19+**  **(n=79)** | **TCRαβ+/CD19+**  **(n=80)** | **P value** |
| --- | --- | --- | --- |
| **Mucositis** | 10 (12%) | 15 (18%) | n.s |
| **Nausea and Vomiting** | 18 (23%) | 32 (40%) | **0.017** |
| **Diarrhea** | 21(26%) | 21 (26%) | n.s. |
| **Hemorrhagic Cystitis** | 22 (27%) | 21 (26%) | n.s. |
| **VOD/SOS** | 2 (2.5%) | 12 (15%) | **0.006** |
| **ES** | 20 (25%) | 16 (20%) | n.s. |
| **TA-TMA** | 9 (11%) | 9 (11%) | n.s |
| **CLS** | 8 (10%) | 11 (14%) | n.s |
| **PRES** | 2 (2.5%) | 3 (4%) | n.s. |
| **Renal toxicity** | 18 (23%) | 16 (20%) | n.s. |

**Abbreviations; TA-TMA: Transplant Associated Thrombotic Microangiopathy. VOD/SOS; Veno-Occlusive Disease/Sinusoidal Obstruction Syndrome; CLS, Capillary Leak Syndrome; PRES, Posterior Reversible Encephalopathy Syndrome; n.s., not significant.**
