# Supplementary material for: T-Cell Depleted Haploidentical Transplantation in Children With Hematological Malignancies: A Comparison Between CD3+/CD19+ and TCRαβ+/CD19+ Depletion Platforms
Source: Front Oncol. 2022 Jun 20;12:884397. doi: 10.3389/fonc.2022.884397 (PMC9251308; doi:10.3389/fonc.2022.884397)
Supplement: Supplementary file 2 [file Table2.docx]

**Supplementary Table S2. Infectious complications**

|  |  |  |  |  |  |
| --- | --- | --- | --- | --- | --- |
| **Infection episodes** | | | **CD3+/CD19+** | **TCRαβ+/CD19+** | **P value** |
|  | | | **(n=149)** | **(n=170)** | **n.s.** |
|  | | |  |  |  |
| **Bacterial infections** | | | **58 (39%)** | **46 (27%)** | **n.s.** |
| **Gram-negative bacilli** | | | **18 (31%)** | **10 (22%)** | **n.s.** |
| *Enterobacteriaceae species* | | | 11 (62%) | 6 (60%) |  |
| *Pseudomonaceae species* | | | 4 (25%) | 3 (30%) |  |
| *Campylobacteraceae species* | | | 3 (13%) | 1 (10%) |  |
| **Gram-positive bacilli** | | | **7 (12%)** | **14 (30%)** | **0.002** |
| *Clostridium species* | | | 7 (100%) | 14 (100%) |  |
| **Gram-positive cocci** | | | **33 (57%)** | **22 (51%)** | **n.s.** |
| *Staphylococcus species* | | | 27(82%) | 17 (77%) |  |
| *Streptococcus species* | | | 5 (15%) | 4 (18%) |  |
| *Enterococcus species* | | | 1 (3%) | 1 (5%) |  |
|  | | |  |  |  |
| **Viral Infections** | | | **77 (52%)** | **108 (63%)** | **0.04** |
| *CMV* | | | 22 (28%) | 28 (26%) |  |
| *Adenovirus* | | | 12 (16%) | 22 (20%) |  |
| *HHV-6* | | | 3 (4%) | 20 (19%) | **0.001** |
| *VZV* | | | 17 (22%) | 10 (9%) | **0.04** |
| *Rinovirus* | | | 4 (5%) | 4 (4%) |  |
| *Coronavirus* | | | 1 (1%) | 2 (2%) |  |
| *RSV* | | | 10 (13%) | 6 (6%) |  |
| *EBV* | | | 3 (4%) | 7 (6%) |  |
| *Other viruses* | | | 5 (7%) | 9 (8%) |  |
| **Fungal Infections** | | | **14 (9%)** | **16 (9%)** | **n.s.** |
| *Candida species* | | | 8 (57%) | 6 (37%) |  |
| *Aspergillus species* | | | 6 (43%) | 8 (50%) |  |
| *Other species* | | | 0 (0%) | 2 (3%) |  |

**Abbreviations; CMV, Cytomegalovirus; HHV-6, Human Herpesvirus 6; VZV, Varicella Zoster Virus; RSV, Respiratory Syncytial Virus;. n.s; not significant**
