# Supplementary material for: T-Cell Depleted Haploidentical Transplantation in Children With Hematological Malignancies: A Comparison Between CD3+/CD19+ and TCRαβ+/CD19+ Depletion Platforms
Source: Front Oncol. 2022 Jun 20;12:884397. doi: 10.3389/fonc.2022.884397 (PMC9251308; doi:10.3389/fonc.2022.884397)
Supplement: Supplementary file 3 [file Table3.docx]

**Supplementary Table S3. Immune cellular reconstitution profile.**

|  | **CD3+/CD19+** | **TCRαβ+/CD19+** | **P value** |
| --- | --- | --- | --- |
| **Day +15** |  |  | **n.s.** |
| **ALC** | 414 (4-3318) | 359 (8-1945) |  |
| **CD3+** | 33 (1-1263) | 52 (0-1122) |  |
| **CD4+** | 15 (0-510) | 10 (0-265) |  |
| **CD8+** | 18 (0-800) | 9 (0-473) |  |
| **NK cells** | 204 (1-1849) | 172 (0-977) |  |
| **B cells** | 0 (0-4) | 0 (0-14) |  |
| **Day +30** |  |  | **n.s.** |
| **ALC** | 591 (52-2083) | 741 (33-6761) |  |
| **CD3+** | 126 (0-1350) | 169 (11-3214) |  |
| **CD4+** | 36 (0-633 | 41 (0-556) |  |
| **CD8+** | 55 (0-930) | 38 (1-1181 |  |
| **NK cells** | 285 (1-1895) | 297 (0-3192) |  |
| **B cells** | 2 (0-245 | 1 (0-414) |  |
| **Day +60** |  |  | **n.s.** |
| **ALC** | 851 (80-5939) | 695 (10-6457) |  |
| **CD3+** | 168 (0-3791 | 194 (3-4746) |  |
| **CD4+** | 91 (0-1166) | 68 (1-370) |  |
| **CD8+** | 92 (0-2862) | 54 (0-7599 | **0.02** |
| **NK cells** | 226 (5-1110) | 275 (3-1451) |  |
| **B cells** | 10 (0-838) | 24 (0-619) |  |
| **Day +90** |  |  | **n.s.** |
| **ALC** | 893 (110-5202) | 823 (51-5323) |  |
| **CD3+** | 279 (0-4242) | 288 (20-2214) |  |
| **CD4+** | 92 (2-1919) | 81 (13-1204) |  |
| **CD8+** | 128 (1-3365) | 91 (7-2546) |  |
| **NK cells** | 212 (13-1100) | 233 (29-1134) |  |
| **B cells** | 35 (0-707) | 118 (0-733) |  |
| **Day +180** |  |  | **n.s.** |
| **ALC** | 1309 (101-3893) | 1262 (82-4843) |  |
| **CD3+** | 590 (39-3123) | 509 (43-8975) |  |
| **CD4+** | 198 (29-881) | 181 (8-901) |  |
| **CD8+** | 280 (6-2390) | 206 (1-2690) |  |
| **NK cells** | 261 (41-1116) | 249 (25-1381) |  |
| **B cells** | 207 (0-2211) | 234 (0-999) |  |
| **Day +270** |  |  | **0.009** |
| **ALC** | 1094 (180-3226) | 2029 (219-4999) |  |
| **CD3+ cells** | 659 (89-5545) | 742 (133-4205) |  |
| **CD4+ cells** | 196 (43-2789) | 341 (20-1692) | **0.05** |
| **CD8+ cells** | 201 (21-2085) | 278 (8-1825) |  |
| **NK cells** | 170 (72-528) | 269 (30-1104) | **0.04** |
| **B cells** | 235 (0-932) | 408 (0-1769) | **0.04** |
| **+ 1 Year** |  |  | **n.s.** |
| **ALC** | 1580 (99-3590) | 1874 (321-4941) |  |
| **CD3+ cells** | 1075 (51-4119) | 994 (114-4352) |  |
| **CD4+ cells** | 350 (34-2550) | 425 (55-2598) |  |
| **CD8+ cells** | 460 (8-1599) | 407 (30-1401) |  |
| **NK cells** | 165 (16-955) | 187 (33-752) |  |
| **B cells** | 308 (0-1040) | 487 (0-1414) |  |
| **+2 Years** |  |  | **n.s.** |
| **ALC** | 2392 (76-5945) | 2500 (801-4120) |  |
| **CD3+ cells** | 1699 (23-4231) | 1583 (426-2596) |  |
| **CD4+ cells** | 741 (17-4308) | 769 (216-1309) |  |
| **CD8+ cells** | 707 (5-2656) | 439 (83-1129) | 0.04 |
| **NK cells** | 141 (34-857) | 214 (43-471) |  |
| **B cells** | 466 (23-1404) | 532 (112-1130) |  |

**Abbreviations, ALC; Absolute lymphocyte count; Lymphocyte subsets are expressed in number of cells/µL**
